# Exploratory dietary patterns: a systematic review of methods applied in pan-European studies and of validation studies

**DOI:** 10.1017/S0007114518001800

**Published:** 2018-08-01

**Authors:** Franziska Jannasch, Fiona Riordan, Lene F. Andersen, Matthias B. Schulze

**Affiliations:** 1 Department of Molecular Epidemiology, German Institute of Human Nutrition Potsdam-Rehbrücke, 14558 Nuthetal, Germany; 2 German Center for Diabetes Research (DZD), 85764 Neuherberg, Germany; 3 Department of Epidemiology and Public Health, University College Cork, Cork T12 K8AF, Republic of Ireland; 4 Department of Nutrition, University of Oslo, 0372 Oslo, Norway

**Keywords:** Systematic literature reviews, Exploratory dietary pattern methods, Dietary patterns, Pan-European studies, DEterminants of DIet and Physical ACtivity knowledge hub, Validation

## Abstract

Besides *a priori* approaches, using previous knowledge about food characteristics, exploratory dietary pattern (DP) methods, using data at hand, are commonly applied. This systematic literature review aimed to identify exploratory methods on DP in pan-European studies and to inform the development of the DEterminants of DIet and Physical ACtivity (DEDIPAC) toolbox of methods suitable for use in future European studies. The search was conducted in three databases on prospective studies in healthy, free-living people across the whole life span. To identify validated DP methods, an additional search without regional restrictions was conducted. Studies including at least two European countries were retained. The search resulted in six pan-European studies applying principal component/factor analysis (PC/FA) (*n* 5) or cluster analysis (*n* 2). The criteria to retain PC/factors ranged from the application of the eigenvalue>1 criterion, the scree plot and/or the interpretability criterion. Furthermore, seven validation studies were identified: DP, derived by PC/FA (*n* 6) or reduced rank regression (RRR) (*n* 1) were compared using dietary information from FFQ (*n* 6) or dietary history (*n* 1) as study instrument and dietary records (*n* 6) or 24-h dietary recalls (*n* 1) as reference. The correlation coefficients for the derived DP ranged from modest to high. To conclude, PC/FA was predominantly applied using the eigenvalue criterion and scree plot to retain DP, but a better description of the applied criteria is highly recommended to enable a standardised application of the method. Research gaps were identified for the methods cluster analysis and RRR, as well as for validation studies on DP.

As people naturally eat a combination of many different foods, the association between single dietary factors and chronic disease risk can be difficult to determine and interpret^(^ ^1^ ^)^. Therefore, methods to investigate dietary patterns associated with morbidity and mortality have gained increasing interest as a complementary approach in nutrition science^(^ ^2^ ^)^. Several systematic literature reviews (SLR) have summarised evidence from studies that investigate the association of dietary patterns with chronic disease risk – for example CVD or type 2 diabetes^(^ ^3^ ^,^ ^4^ ^)^. Alongside *a priori* approaches, which use preliminary knowledge about the detriment or benefit of certain foods for a health outcome, exploratory approaches – using data at hand without any previous hypothesis – have been commonly applied to derive dietary patterns. Examples for exploratory approaches are factor analysis and principal component analysis (PCA), which use the covariance matrix of the food groups to reduce the dimensionality from a high number of food groups to few patterns of food consumption^(^ ^5^ ^)^. While principal components are linear combinations of the observed variables, factors derived by factor analysis can be understood as latent constructs^(^ ^6^ ^)^. Another exploratory approach is cluster analysis, which groups participants with similar dietary habits instead of correlated food groups^(^ ^7^ ^)^. Contrary to factor analysis and PCA, where study participants can belong to more than one factor or principal component, cluster analysis groups participants into mutually exclusive, non-overlapping clusters^(^ ^8^ ^)^. The mixed approaches, reduced rank regression (RRR) and partial least square method, also use the covariance matrix of the food groups and combine this with previous knowledge about nutrients or biomarkers, which are involved in the development of a certain health outcome^(^ ^9^ ^)^.

In comparison with *a priori* indices, which are applicable across different study populations, exploratory approaches result in population-specific dietary patterns, because these methods are exclusively based on data at hand. In particular, in investigations including different populations with likely differences in culinary habits, as can be expected, for example, in pan-European investigations, the population specificity of exploratory dietary patterns constitutes a challenging task, because differences in food intake distributions lead to heterogeneous dietary pattern compositions. With regard to methodological considerations, exploratory approaches require several decisions to come to a final solution of factors or clusters, and partially subjective decisions cannot be ruled out.

So far, to our knowledge, no systematic investigation of methodological characteristics of exploratory dietary pattern approaches related to investigations in the context of multi-centre or multi-country studies has been conducted. In the Framework of the Joint Programming Initiative ‘A Healthy Diet for a Healthy Life’ within the EU Committee, the DEterminants of DIet and Physical ACtivity (DEDIPAC) Knowledge Hub has been developed. This was a European transdisciplinary research network programme, which aimed to realise a more effective promotion of healthy diets and physical activity. Therefore, it aimed to identify state-of-the-art methods, enabling future cross-country interventions and policies^(^ ^10^ ^)^. Within this framework, this systematic literature review specifically aimed to identify and compare (validated, if possible) exploratory dietary pattern methods, which were conducted in pan-European studies in order to deduce recommendations for such analyses in pan-European settings and beyond and to identify potential research gaps.

## Methods

### Data sources and study selection

A detailed plan for the conduction of the systematic review of pan-European studies was established in advance, and the respective protocol for the SLR can be accessed from PROSPERO (CRD42014014318). A systematic literature search was conducted in the databases MEDLINE, Web of Science and Embase, which encompassed search terms covering different thematic areas. The first area was described by terms that depict dietary habits or patterns. These were linked to the second area of *a posteriori* statistical methods. Terms for *a priori* methods were also included in the search to identify studies that might have applied several dietary pattern approaches. As one aim was to detect pan-European studies, which means that they were conducted in more than two European countries, the search included the names of European countries, as well as terms that addressed the multi-country aspect. The language was restricted to English. To ensure the inclusion of studies whose focus was on humans, animal studies were excluded. Owing to the relatively recent application of the *a posteriori* approaches on nutrition data, the search was limited to literature published between 1 January 1990 and 15 January 2018. Details of the search strategy can be found in the online Supplementary Table S1.

The screening of titles and abstracts of the identified articles was conducted by F. J. and F. R. independently. If doubts occurred, which could not be resolved, the article was retained for the next screening step. Any disagreement during the final full-text review stage was resolved through discussion of the articles concerned. No exclusions were made regarding any age group, sex, socio-economic status or ethnicity, but the study populations were required to be free-living and healthy. In addition, no restriction was set to the study design. Additionally, reference lists of the identified articles and reviews, which seemed to be relevant, were screened for a comprehensive overview. No ‘grey literature’ – that is conference papers or unpublished manuscripts – were included in this SLR. In case of the DIETSCAN study and European Prospective Investigation into Cancer and Nutrition (EPIC)-Elderly^(^ ^11^ ^,^ ^12^ ^)^, which were identified several times^(^ ^13^ ^–^ ^15^ ^)^, the publications with the most detailed dietary pattern method^(^ ^11^ ^,^ ^13^ ^)^ were retained for further consideration.

### Data extraction and quality assessment

Data extraction was done by one reviewer and confirmed by a second reviewer. An excel sheet, which captured all relevant information to answer the research question, was developed and included the following data: title, author, year, name and design of the study, countries and their contributing sample size and follow-up time. Details of the study population were also extracted: for example, sex, age, ethnicity, socio-economic status and educational level. The dietary assessment method, the statistical method to derive dietary patterns, the pattern label and variation between different study populations were considered as relevant.

As the search was not limited to a distinct health outcome, quality assessment tools such as the SIGN checklist (developed by Scottish Intercollegiate Guidelines Network) were not applicable. A more general quality assessment was developed, which included five questions and a formula to calculate a score. Possible answers were scored as follows: ‘yes’ (2 points), ‘partially’ (1 point), if at least some information was provided, ‘no’ (0 points) (online Supplementary Table S2):

(1) Is the design evident to answer our study question?
(2) Are the subject characteristics sufficiently described?
(3) Is the method of diet assessment described?
(4) Is the diet pattern method well defined and are the details of assessment reported?
(5) Is some estimate of variance reported for the dietary patterns?

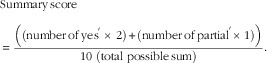



### Additional search for validation studies

Notwithstanding the PROSPERO protocol, an additional search strategy (online Supplementary Table S3) was developed and the search was conducted on 1st July 2016 in the databases Medline and Web of Science to identify studies that validated dietary patterns, because none of the identified studies according to the main aim of the SLR focused on the validation of the derived dietary patterns. As this SLR aims to give an overview of methodological considerations of exploratory statistical approaches, the additional search was not limited to pan-European studies.

## Results

### Description of the included studies

**Fig. 1:**
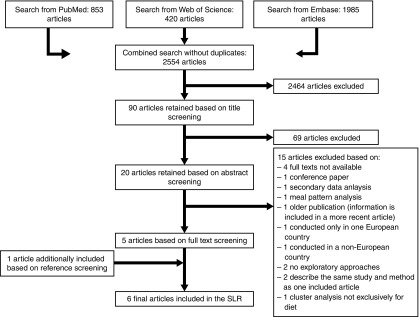
Flow diagram of the article screening process. SLR, systematic literature review.

The initial search identified 2816 articles, which resulted in 2554 articles after removing all duplicates. Titles and abstracts were screened regarding the inclusion criteria, and the full-text screening comprised twenty articles (Fig. 1), which resulted in five articles being retained^(^ ^11^ ^,^ ^16^ ^–^ ^19^ ^)^. On the basis of the reference screening of the remaining articles, one additional publication^(^ ^13^ ^)^ was included for data extraction, resulting in a total of six final articles.

The main characteristics of the included studies are described in Table 1. The number of included European countries ranged from four^(^ ^13^ ^)^ to twelve^(^ ^17^ ^)^. The number of participants ranged from small studies with 807 participants^(^ ^16^ ^)^ to larger studies with 107 673 participants^(^ ^13^ ^)^. One study investigated dietary patterns in children^(^ ^19^ ^)^, two studies in participants aged over 60 or 70 years (EPIC-Elderly^(^ ^11^ ^)^ and SENECA^(^ ^16^ ^)^), whereas the other studies^(^ ^13^ ^,^ ^17^ ^,^ ^18^ ^)^ investigated dietary patterns in adults aged between 35 and 76 years. Information about the socio-economic status of participants was reported in one included publication^(^ ^17^ ^)^ and education as an important characteristic of the study population was described in two publications^(^ ^11^ ^,^ ^17^ ^)^. In the INTERHEART study^(^ ^17^ ^)^, education was considered as part of the socio-economic status.

**Table 1:** Summary of the included studies (*n* 6) and their characteristics

Author/study (year)	Study design	Population	Countries	Age	Ethnicity	SES	Education
Balder/DIETSCAN project including: ATBC NLCS SMC ORDET (2003)^(^ ^13^ ^)^	ATBC: randomised placebo-controlled intervention study NLCS: prospective cohort study SMC: population-based mammography screening ORDET: prospective cohort study	ATBC: 27 111 men NLCS: 3123 (1525 men and 1598 women) SMC: 66 651 women ORDET: 10 788 women	Finland The Netherlands Sweden Italy	ATBC: 50–69 years NLCS: 55–69 years SMC: 40–76 years ORDET: 35–69 years	No information	No information	No information
Bamia/EPIC-Elderly study (2005)^(^ ^11^ ^)^	Prospective cohort study	74 607 from all EPIC cohorts except Norway (too young)	UK Germany The Netherlands Spain France Denmark Sweden Greece Italy	≥60 years	No information	No information	Level of educational achievement: no/primary school technical school secondary school university degree
Havemann-Nies/SENECA (1998)^(^ ^16^ ^)^	Survey from 1993	379 men and 428 women	France Italy The Netherlands Switzerland Poland	Born between 1913 and 1918	No information	No information	No information
Iqbal/INTERHEART study (2008)^(^ ^17^ ^)^	Case–control study	Total: 12461 incident cases of AMI; 14637 controls free of any heart disease (no information about individual contribution)	52 countries around the world (for Europe including): Croatia Czech Republic Germany Greece Hungary Italy The Netherlands Poland Portugal Spain Sweden UK	No information	No information	SES by household income (range 1–5) and education (no education, grades 1–8, grades 9–12, trade school, university/college)	Education included in SES
Menotti/Seven Countries Study (1999)^(^ ^18^ ^)^	Prospective cohort study	12 763 men in total	Finland Italy Greece (Former Yugoslavia) Japan USA Serbia The Netherlands	40–59 years at baseline	No information	No information	No information
Pala/IDEFICS (2013)^(^ ^19^ ^)^	Prospective cohort study	9427 in total: 1521 Italy 1251 Estonia 1049 Cyprus 1111 Belgium 1358 Sweden 1010 Germany 991 Hungary 1136 Spain	Belgium Cyprus Estonia Germany Hungary Italy Spain Sweden	2–9 years	No information	No information	No information

SES, socio-economic status; DIETSCAN, Dietary Patterns and Cancer; ATBC, Alpha-Tocopherol, Beta-Carotene Cancer Prevention Study; NLCS, Netherlands Cohort Study; SMC, Swedish Mammography Cohort; ORDET, Hormones and Diet in the Etiology of Breast Cancer Risk; EPIC, European Prospective Investigation into Cancer and Nutrition; AMI, acute myocardial infarction; IDEFICS, Identification and prevention of Dietary- and lifestyle-induced health EFfects In Children and infantS.

The exploratory approaches used to generate dietary patterns in the identified pan-European studies were limited to two. Factor analysis^(^ ^17^ ^,^ ^18^ ^)^ or PCA^(^ ^11^ ^,^ ^13^ ^,^ ^19^ ^)^ was applied in five studies (Table 2), whereas cluster analysis was applied in two studies^(^ ^11^ ^,^ ^16^ ^)^ (Table 3). For those studies that applied factor analysis or PCA, the number of identified patterns ranged from one pattern in the Seven Countries Study^(^ ^18^ ^)^ and EPIC-Elderly^(^ ^11^ ^)^ to seven patterns in the DIETSCAN study^(^ ^13^ ^)^. Labels were given to patterns according to the food groups that characterised the patterns – for example, the ‘pork, processed meat and potatoes’ pattern or ‘(salad) vegetables’ pattern in the DIETSCAN study^(^ ^13^ ^)^ or the ‘plant-based’ dietary pattern in the EPIC-Elderly study^(^ ^11^ ^)^. Another approach to labelling the patterns involved description of regional dietary habits such as the ‘oriental’ or ‘western’ pattern in the INTERHEART study^(^ ^17^ ^)^. In the Identification and prevention of Dietary- and lifestyle-induced health EFfects In Children and infantS (IDEFICS) study^(^ ^19^ ^)^, the pattern labels were a mixture of eating behaviour (snacking), food groups characterising the pattern (‘vegetables and whole meal’) and macronutrients (‘protein and water’). The Seven Countries Study^(^ ^18^ ^)^ did not use any label for the single derived pattern. In the SENECA study, where exclusively snack food was considered, five clusters were labelled with regard to food groups that characterised them^(^ ^16^ ^)^. In EPIC-Elderly, three clusters were identified and labelled A, B and C^(^ ^11^ ^)^.

**Table 2:** Overview of the studies using factor analysis or principal component analysis (PCA) to derive dietary patterns (DP)

Author/study (year)	Diet assessment instrument	Details of diet assessment instrument	Reported DP method	Details of DP method	Label of DP	Variation of DP across regions/countries	Reliability/validity (yes/no)
Balder/DIETSCAN project including: ATBC NLCS SMC ORDET (2003)^(^ ^13^ ^)^	Country-specific validated dietary instruments	Aggregation of the four FFQ data to 51 common food groups (including also country-specific foods)	Exploratory factor analysis	Sensitivity analyses; decision for extraction: eigenvalue>1 and scree plot; dichotomised variables with>75 % non-users (non-user=0, user=1); no transformations to enhance linearity or normality; no exclusion of outliers, because of intensive data cleaning; factor loadings>0·35 considered	Factors labelled: (salad) vegetables; pork, processed meat, potatoes; cooked vegetables; alcohol; sweet and/or savoury snacks; brown/white bread substitution; others	Vegetables and meat pattern for all NLCS men: cooked vegetables, sweet/savoury snacks, bread substitution NLCS women: sweet/savoury snacks, bread substitution, fat dairy SMC: alcohol, margarine/butter substitution ORDET: cooked vegetables, alcohol	No internal validity via several sensitivity analyses
Bamia/ EPIC-Elderly study (2005)^(^ ^11^ ^)^	Country-specific dietary self-reported or interviewer-administered questionnaires	22 condensed energy-adjusted food groups (residual method); validated questionnaires	PCA	Number of PC retained by three criteria: eigenvalue exceeding 1, scree plot, interpretability of each component	Plant-based DP	Greece, Italy, Spain and France highest proportion in the third tertile; Sweden, Denmark low scores; Germany, The Netherlands and UK highest proportion in the second tertile	No
Iqbal/INTERHEART study (2008)^(^ ^17^ ^)^	19-item qualitative food group frequency questionnaire (already condensed food items)	Generic questionnaire to be applicable in multiple countries; no portion size, only frequency; standardised in consumption per day	Exploratory factor analysis	Rotated orthogonally; retain factors with eigenvalue>1; scree test, factor interpretability (not the percentage of variance)	Oriental pattern: tofu and soy and other sauces; Western pattern: fried food, salty snacks, and meat intake; prudent pattern: fruit and vegetable intake	Western and central Europe: highest adherence to prudent pattern, followed by western and oriental pattern	No
Menotti/Seven Countries Study (1999)^(^ ^18^ ^)^	7-d record (14 of 16) 1 d record (USA) 4 d record (Japan)	18 food groups classified in all cohorts: bread, cereals, potatoes, vegetables, legumes, fruits, sugar, oils, butter, meat, fish, eggs, margarine + lard, milk, cheese, pastries, alcohol, and ‘other’; some analyses run on combinations of the 18 food groups: ‘vegetables foods’, ‘animal foods’, ‘sweets'	Factor analysis	No information	1 factor score	Highest factor score and highest risk for CHD: east and west Finland, the Netherlands, Serbia; lowest factor and lowest risk for CHD: Italy and Greece	No
Pala/IDEFICS (2013)^(^ ^19^ ^)^	CEHQ	Reproducible and validated; completed by parents or other caregivers (asks about consumption in preceding month)	PCA	Kaiser–Meyer–Olkin sampling adequacy was>0·6, which supports use of PCA; criteria for retained DP: eigenvalue, scree plot, factor interpretability), factor loadings>0·2 considered; for stability, also DP from the follow-up sample assessed; simplified DP (food variables with high loadings were standardised and summed)	Component 1 (Snacking) Component 2 (sweet and fat) Component 3 (vegetables and wholemeal) Component 4 (protein and water)	No information	Reliability assessed by generating DP in the subgroup of the follow-up participants and comparing it with the original DP

DIETSCAN, Dietary Patterns and Cancer; ATBC, Alpha-Tocopherol, Beta-Carotene Cancer Prevention Study; NLCS, Netherlands Cohort Study; SMC, Swedish Mammography Cohort; ORDET, Hormones and Diet in the Etiology of Breast Cancer Risk; EPIC, European Prospective Investigation into Cancer and Nutrition; IDEFICS, Identification and prevention of Dietary- and lifestyle-induced health EFfects In Children and infantS; CEHQ, Children's Eating Habits Questionnaire.

**Table 3:** Overview of the studies using cluster analysis to derive dietary patterns (DP)

Author/study (year)	Diet assessment instrument	Details of diet assessment instrument	Reported DP method	Details of DP method	Label of DP	Variation of DP across regions/countries	Validity
Havemann-Nies/SENECA (1998)^(^ ^16^ ^)^	3-d estimated record and frequency checklist of food groups	By trained personnel; country-specific food composition tables	Cluster analysis	Ward’s minimum variance method: number of clusters chosen on the basis of *R* ^2^ and the composition of the clusters	‘Light snackers’ ‘Fruit and vegetables’ ‘Snackers’ ‘Sweet drinkers’ ‘Dairy snackers’ ‘Alcohol’ drinkers	Clusters present in all towns; dairy snackers almost all in Culemborg; alcohol drinkers almost all in Haguenau	No
Bamia/EPIC-Elderly (2005)^(^ ^11^ ^)^	Country-specific dietary self-reported or interviewer-administered questionnaires	22 condensed energy-adjusted food groups (residual method); validated questionnaires	Cluster analysis	Ward’s minimum variance method: pseudo *F*, pseudo *t* ^2^, cubic clustering criterion, tree diagram	Cluster A Cluster B Cluster C	Cluster A predominant in Italy, Spain and Greece; Cluster B and C fairly distributed in France and northern Europe; Denmark exclusively in Cluster C	No

EPIC, European Prospective Investigation into Cancer and Nutrition.

Four of the six identified studies achieved a good appraisal with nine to ten quality points. The remaining two studies achieved a moderate appraisal with seven points (online Supplementary Table S2).

### Methods to generate dietary patterns

#### Details of the dietary assessment instruments

The most commonly used dietary assessment instrument throughout the studies was the FFQ (Table 2). In most publications, information was available on the number of food groups, which were frequently condensed from a larger number of food items implemented in the questionnaires. For example, in the DIETSCAN project^(^ ^13^ ^)^ the FFQ from the different cohorts included sixty-seven to 276 single food items, which were aggregated to fifty-one common food groups to be defined as having a specific role in the diet and a possible relevance to the aetiology of the health outcome. In contrast to that, investigators of the INTERHEART study^(^ ^17^ ^)^ used a generic questionnaire applicable in multiple countries with a small number of preselected food groups (*n* 19). To assess dietary habits in 2- to 9-year-old children in the IDEFICS study^(^ ^19^ ^)^, the Children’s Eating Habits Questionnaire was developed, comprising forty-three food groups, which was provided in local languages and with additional explanations for the parents or other caregivers. In EPIC-Elderly, dietary intake was mainly assessed with FFQ, partly also semi-quantitative FFQ, which were characterised by country-specific components. Subsequently, twenty-two food groups were aggregated, which were comparable among all participating EPIC centres^(^ ^11^ ^)^. In the Seven Countries Study^(^ ^18^ ^)^, dietary records were used to capture the dietary intake: in fourteen of sixteen countries 7-d records were applied, except in the USA (1-d record) and Japan (4-d record). The food items were then summarised into eighteen food groups. In the SENECA study, participants were asked to record their food intake with 3-d dietary records^(^ ^16^ ^)^. As this study aimed to investigate snack patterns, information about eating occasions was collected and a selection of fifteen food groups was considered as snack foods.

Three studies^(^ ^11^ ^,^ ^13^ ^,^ ^19^ ^)^ reported to use validated dietary assessment instruments, whereas one study stressed that only face validity of the instrument was investigated^(^ ^17^ ^)^. However, no information on the validity or reliability of the assessment instruments was provided in the other two studies^(^ ^16^ ^,^ ^18^ ^)^.

#### Details of the identification of dietary patterns

The aim of exploratory statistical approaches is the identification of certain underlying structures of dietary intake. Although those approaches are exclusively data-based, decisions to identify these dietary patterns remain arbitrary. Three studies reported that factor analysis was applied^(^ ^13^ ^,^ ^17^ ^,^ ^18^ ^)^. However, the criteria to identify the dietary patterns were specifically described for PCA. In one study^(^ ^19^ ^)^, the Kaiser–Meyer–Olkin criterion was applied to investigate the adequacy of sampling of the food groups used in the PCA. From the five identified studies applying PCA, three studies^(^ ^11^ ^,^ ^17^ ^,^ ^19^ ^)^ used the criteria eigenvalue>1, scree plot and interpretability of the principal components to decide upon the number of dietary patterns to retain, whereas one study^(^ ^13^ ^)^ solely used the first two criteria. Menotti *et al*.^(^ ^18^ ^)^ did not report any criteria. The eigenvalue>1 criterion implies that only those principal components with an eigenvalue>1 will be retained. Considering that the eigenvalue is the amount of variance accounted for by one principal component and each observed food variable contributes one unit of variance to the total variance, principal components with eigenvalues>1 represent a data reduction^(^ ^6^ ^)^. Nevertheless, with this criterion the number of retained factors can be quite large. Therefore, a plot of the eigenvalues of principal components (scree plot) may help to decide the final number of retained principal components, by visually distinguishing a small number of components, which explain a lot of variance in the food groups, from the residual components, which explain a minor amount of variance^(^ ^6^ ^)^. Besides these two criteria, investigators frequently took into account the interpretability of identified patterns. Interpretability usually considers the conceptual meaning of an identified principal component, but in terms of dietary patterns it is difficult to adjudicate on a combination of food groups. Furthermore, in the articles, where interpretability was listed as a criterion, no explanations were given as to what was meant by this. Although the retained principal components consisted of all original food groups, cut-offs for the factor loadings of food groups were frequently set to identify those foods that were meaningful contributors to the pattern. This cut-off ranged between factor loadings of 0·20^(^ ^19^ ^)^ and 0·35^(^ ^13^ ^)^ in the identified studies. In two articles^(^ ^11^ ^,^ ^13^ ^)^, the food groups were energy-adjusted with the residual method, developed by Willett and Stampfer^(^ ^20^ ^)^, before they were included in the analyses to account for total energy intake.

The second identified exploratory method, namely cluster analysis, was applied in two pan-European studies^(^ ^11^ ^,^ ^16^ ^)^. Both studies used the Ward’s minimum variance method but different criteria to derive the final cluster solution. In general, two clustering techniques for deriving dietary patterns can be distinguished: the hierarchical and non-hierarchical clustering. As a hierarchical approach of clustering people according to their dietary habits, the Ward’s minimum variance method was applied in the identified study^(^ ^16^ ^)^. This is an agglomerative method, starting with each observation as its own cluster and merging together to a larger cluster^(^ ^21^ ^)^. For that purpose, the ANOVA, retaining only those pairs of clusters with the smallest increase in the error sum of squares, is usually used^(^ ^22^ ^)^. The number of cluster solutions was chosen based on the proportion of the explained variance of all variance (R^2^) of the clusters and on their composition^(^ ^16^ ^)^.

#### Validity of dietary patterns

Although the included studies partly assessed the validity of the dietary assessment instrument^(^ ^11^ ^,^ ^19^ ^)^, none of the identified pan-European studies has yet investigated the validity of dietary patterns. However, existing attempts at validation, which were not limited to the pan-European context, were identified with an additional systematic literature search (online Supplementary Table S3). Seven studies could be included^(^ ^23^ ^–^ ^29^ ^)^ (Table 4). Of these, two studies investigated dietary patterns in adolescents^(^ ^23^ ^,^ ^24^ ^)^, whereas adults were investigated in the other five studies^(^ ^25^ ^–^ ^29^ ^)^. Six published articles validated dietary patterns derived by factor analysis^(^ ^23^ ^,^ ^25^ ^,^ ^27^ ^)^ or PCA^(^ ^26^ ^,^ ^28^ ^,^ ^29^ ^)^ and one study by RRR^(^ ^24^ ^)^, respectively. The dietary assessment instruments to measure food intake were predominantly FFQ, which were validated in the majority of studies^(^ ^23^ ^,^ ^24^ ^,^ ^26^ ^)^. In the Japanese study, a diet history questionnaire referring to the previous month was used^(^ ^29^ ^)^. Dietary records mostly served as validation instruments, but the recording methods differed with regard to the time frame from 3 d^(^ ^23^ ^,^ ^24^ ^)^, 4 d^(^ ^29^ ^)^ to 1 week^(^ ^26^ ^,^ ^27^ ^)^. In the study by Asghari *et al*.^(^ ^25^ ^)^, the mean of twelve 24-h dietary recalls was applied. The frequency of application ranged from one time^(^ ^23^ ^,^ ^24^ ^)^ to four times^(^ ^29^ ^)^ for dietary records. Dietary patterns derived by PCA/factor analysis were either retained by using the scree plot^(^ ^26^ ^,^ ^29^ ^)^, the eigenvalue and scree plot^(^ ^24^ ^)^ or all three common criteria (including interpretability as an additional criterion)^(^ ^26^ ^,^ ^28^ ^)^. In the study by Khani *et al*.^(^ ^27^ ^)^, only those principal components with eigenvalues >1·8 were retained. The authors reported to use the same criteria for deriving the dietary patterns, except for Ambrosini *et al*., who applied different eigenvalue criteria (>1 for FFQ-derived dietary data, <1 for food record-derived data)^(^ ^23^ ^)^. The retained dietary patterns resulted in similar numbers and comparable compositions, when patterns derived by the study instrument were validated against patterns derived by the reference instrument (Table 4). The correlation coefficients between the derived scores were modest in all included studies. Ambrosini *et al*.^(^ ^23^ ^)^ reported higher correlation coefficients if energy-adjusted dietary patterns were used. The percentage of the variance explained in dietary patterns using data from dietary records was higher than from FFQ in two studies^(^ ^25^ ^,^ ^27^ ^)^, whereas it was comparable in the study by Okubo *et al*.^(^ ^29^ ^)^. The dietary patterns derived by RRR using data from an FFQ and from a food record were similar in composition, and modest agreement between the dietary pattern scores was observed^(^ ^24^ ^)^.

**Table 4:** Overview of included studies that validated their dietary patterns

Studies	Population size	Sex/age range	Study instrument/time frame	Reference instrument/time frame	Reported dietary pattern method	Correlation	Limits of agreement	Further assessment
PCA/FA								
Western Australian Pregnancy Cohort (Raine) Study^(^ ^23^ ^)^	Total *n* 3195 FFQ *n* 2337 FR *n* 858	Male and female/14 years	212-item FFQ/previous year	1×FR/3 d	FA	Crude: Healthy: *r* 0·43 Western: *r* 0·27 Energy adjusted: Healthy: *r* 0·45 Western: *r* 0·36 Deattenuated: n.a.	Healthy: 0·03 (−1·69–1·75) Western: −0·03 (−1·89–1·82)	
Tehran Lipid and Glucose Study^(^ ^25^ ^)^	Total *n* 132 Males *n* 61 Females *n* 71	Male and female 20–70 years	168-item FFQ/previous year	12×dietary recall/ previous 24 h	FA	Crude: Iranian traditional: *r* 0·48 Western: *r* 0·74 Energy adjusted: n.a.Deattenuated: Iranian traditional: *r* 0·48 Western: *r* 0·75		
Health Professionals Follow-up study^(^ ^26^ ^)^	Total *n* 127	Male/40–75 years	131-item FFQ (FFQ1 and FFQ2)/previous year	2×DR/7 d	PCA	Crude: Prudent: DR *v*. FFQ1: *r* 0·34 DR *v*. FFQ2: *r* 0·41 Western: DR *v*. FFQ1: *r* 0·51 DR *v*. FFQ2: *r* 0·64 Energy adjusted: n.a.Deattenuated: Prudent: DR *v*. FFQ1: *r* 0·45 DR *v*. FFQ2: *r* 0·52 Western: DR *v*. FFQ1: *r* 0·58 DR *v*. FFQ2: *r* 0·74		
SMC^(^ ^27^ ^)^	Validation study *n* 129, reproducibility study *n* 212	Female/40–74 years	60-item FFQ/previous year	4×DR/7 d	FA	Crude: Healthy: *r* 0·47 Western: *r* 0·41 Drinker: *r* 0·73 Energy adjusted: n.a.Deattenuated: Healthy: *r* 0·59 Western: *r* 0·50 Drinker: *r* 0·85		
Japan Public Health Center-based Prospective Study^(^ ^28^ ^)^	Total *n* 498 Males *n* 244 Females *n* 254	Male and female/ 56, 59 years	138-item FFQ/previous year	4×or 2×DR/ 28 or 14 d	PCA	Crude: n.a. Energy-adjusted: Men: Prudent: *r* 0·47 Westernised: *r* 0·32 Traditional: *r* 0·49 Women: Prudent: *r* 0·36 Westernised: *r* 0·56 Traditional: *r* 0·63 Deattenuated: n.a.		
Three areas in Japan: Osaka (urban), Nagano (rural inland) and Tottori (rural coastal)^(^ ^29^ ^)^	Male *n* 92 Female *n* 92	Male and female 30–69 years	4×145-item DHQ/previous month	4×weighed DR/4 d	PCA	Crude: n.a. Energy-adjusted: Women: Healthy: *r* 0·57 Western: *r* 0·44 Japanese traditional: *r* 0·44 Men: Healthy: *r* 0·62 Western: *r* 0·56 Deattenuated: Women: Healthy: *r* 0·63 Western: *r* 0·45 Japanese traditional: *r* 0·69 Men: Healthy: *r* 0·65 Western: *r* 0·53	Women: Healthy: −1·81–+1·81 Western: −2·22–+2·22 Japanese traditional: −2·08–+2·08 Men: Healthy: −1·83–+1·83 Western: −1·71–+1·71	
Mixed approaches								
Western Australian Pregnancy Cohort (Raine) Study^(^ ^24^ ^)^	Total *n* 783	Male and female/ 14 years	227-item FFQ/previous year	1×FR/ 3 d	RRR	Crude: Girls: *r* 0·35 Boys: *r* 0·49 Energy adjusted: n.a.Deattenuated: n.a.	Girls: −0·08 (95 % CI −0·21, 0·04) Boys: −0·05 (95 % CI −0·17, 0·07)	Bland–Altman plots

PCA, principal component analysis; FA, factor analysis; FR, food record; n.a., no analysis; DR, dietary record; SMC, Swedish Mammography Cohort; DHQ, diet history questionnaire; RRR, reduced rank regression.

## Discussion

Two exploratory dietary pattern approaches were identified in pan-European studies: PCA/factor analysis and cluster analysis. Although factor analysis and PCA are conceptually different, strong similarities in their application using standard statistical software made it, in many cases, unclear as to which method was indeed used by the included studies. We therefore discuss such studies together. In studies that applied PCA/factor analysis to derive dietary patterns, two criteria to select dietary patterns (eigenvalue, scree plot) were commonly applied, whereas the third criterion of interpretability was applied in half of the studies. As the latter criterion was insufficiently described, it highlights the demand for a better reporting of what is exactly meant by ‘interpretability’ to enhance transparency and enable the replication of pattern methods. The described three criteria have also been applied in numerous single-country studies, as summarised in recent comprehensive SLR^(^ ^3^ ^,^ ^4^ ^)^. Hence, they appear to be commonly applied methods. As we aimed to deduce recommendations for future investigations, the quality of the included studies was investigated to ensure best practice methods. We developed our own quality rating system to consider important criteria such as the sufficient reporting of the dietary assessment and dietary pattern approach. Common quality rating systems rather take into account risk assessments between dietary exposures and the development of a certain disease outcome, which was not the focus of this review. Furthermore, information on validated dietary assessment instruments was also part of our quality rating, as this could determine the overall quality of the study. Although four of the six studies were rated good, two studies were rated moderate owing to their lack of using validated assessment instruments.

Two pan-European studies used cluster analysis to derive dietary patterns. Both applied Ward’s minimum variance method, but reported different criteria to come to a final cluster solution. To put it in a wider context, a review published by Devlin *et al*.^(^ ^8^ ^)^ stated that cluster analysis techniques are highly influenced by researcher bias, for example the decision for the final number of clusters, as there is no standard available. A comparison of three cluster analysis methods, namely Ward’s minimum variance method, k-means procedure and flexible-beta method, was undertaken by Lo Siou *et al*.^(^ ^22^ ^)^ and it was concluded that clusters derived by the k-means procedure were more reproducible than the other two methods. However, the authors also concluded that it is likely that other methods would be more appropriate as data reduction techniques, but up to now reviews identified the *k*-means procedure as the most commonly applied method^(^ ^7^ ^,^ ^8^ ^)^.

In the context of pan-European investigations, the standardisation of dietary assessment and dietary data processing with regard to the availability of food items across different countries (either included in a specific questionnaire or later condensed from a higher number of assessed food items) constitutes an important part of the methodology. Nevertheless, this can potentially be at the expense of country-specific food choices, which could mean a possible loss of relevant details in the determination of a diet–disease relationship^(^ ^13^ ^)^. This issue could be also extrapolated as relevant to other multi-country comparisons or even single-country multi-centre studies, when differences in dietary habits and culinary usage are present. Regarding the actual dietary pattern composition, it is deemed necessary to evaluate whether this pattern is applicable across several countries or whether it rather reflects country-specific differences. Indeed, the majority of studies investigated this issue and three studies^(^ ^11^ ^,^ ^17^ ^,^ ^18^ ^)^ identified rather region-specific patterns. In contrast, Balder *et al*.^(^ ^13^ ^)^ identified in all countries the first two components with comparable compositions, whereas the components 3 to 5 rather reflected country-specific patterns.

Besides these considerations, the impact of transforming dietary data before exploratory patterns analyses – for example by different forms of energy-adjustment of food groups – on pattern solutions has not been sufficiently evaluated yet. In one study^(^ ^13^ ^)^, the authors concluded that these considerations did not materially change the number of factor solutions, but resulted in patterns stronger reflecting food substitution mainly for those dietary patterns, which had high contributions of energy-dense food groups. Clearly, more research is needed to also clarify this question and enable definite recommendations how to control for total energy intake.

With regard to the pan-European context, no study has been conducted yet on deriving dietary patterns by RRR. However, this presents quite a challenge, as not only information on food groups needs to be available across several countries but information is also required on specific biomarkers or nutrients, which can be linked to the respective health outcome of interest. Furthermore, results have to be interpreted with caution, because the two steps of deriving this disease-specific dietary pattern and relating it to the disease can lead to overoptimistic results if investigated within the same study population. Therefore, it is highly recommended to evaluate the results in an external study population to test for generalisability^(^ ^9^ ^)^, as it was done in several existing studies for type 2 diabetes^(^ ^30^ ^–^ ^32^ ^)^.

As it was already concluded in our previous systematic review, the application of exploratory methods results in population-specific dietary patterns, which were highly heterogeneous across populations^(^ ^4^ ^)^. Also in this review, the identified dietary patterns largely differed in their composition and labelling by the authors. To our knowledge, exploratory pattern approaches have not been tested with regard to validity and reliability in the pan-European context so far. However, we identified several validation studies not limited to pan-European studies with an additional search. Summarising from this set of studies, those dietary patterns that were derived by PCA/factor analysis had a rather modest validity when compared with the respective reference instruments. This could be constituted by methodological differences between dietary assessment methods. Although an FFQ better captures episodically eaten foods owing to a longer reported time span, a dietary record usually reports a smaller range of food groups, probably providing excess zero consumption and therefore an underestimation of the usual consumption of certain food groups. However, this is largely depending on the frequency of application and hence on the comprised time span^(^ ^23^ ^,^ ^29^ ^,^ ^33^ ^)^. Consequently, partly different dietary pattern structures, hence lower correlation coefficients, could occur. However, attenuating the validity is not the only concern. If the investigation of the relative validity of dietary patterns was solely restricted to food groups that were assessed in both the study and reference dietary assessment instruments, then it is important to consider that this could potentially result in an overoptimistic validity^(^ ^29^ ^)^. Regarding the criteria to retain a certain number of principal components/factors, no standard could be deduced, because the identified studies largely differed in their application of respective criteria. We identified only one study that investigated the relative validity of RRR patterns from an FFQ against a dietary record in adolescents and observed a modest agreement^(^ ^24^ ^)^.

Strength of this SLR was the comprehensive search in three distinct databases and an additional reference screening to identify all existing publications, which offered information on dietary patterns in the pan-European context. Nevertheless, it could not be ruled out that we did not identify all relevant approaches. A further strength was the measurement of the quality of the included studies, although no commonly used checklist (e.g. SIGN checklist) was applied. However, we captured several aspects of quality assessment by determining five questions, which were formulated to identify high-quality studies for the specific aim of this SLR.

### Conclusion

To conclude, the literature search identified six studies that applied exploratory statistical approaches to derive dietary patterns in the pan-European context. PCA/factor analysis was the predominant approach and the eigenvalue>1 and scree plot were the most commonly applied criteria to decide upon the number of principal components/factors to retain. Nevertheless, a more detailed description and justification for the applied method (PCA *v*. factor analysis) and criteria, particularly the interpretability criterion, is demanded to ensure a better comparability of the actual applied methods. Clear gaps were identified for cluster analysis that was applied in two studies, where criteria vigorously differed, although both studies reported to use Ward’s minimum variance method. Approaches such as RRR have not yet been applied in pan-European studies. Concluding from an additional search of validation studies, moderately correlated dietary patterns were identified, which did not alter in the number and composition of dietary patterns when PCA/factor analysis was applied on intake data from different dietary assessment instruments. Nevertheless, it is highly recommended to investigate the validity of dietary patterns across countries to ensure a certain generalisability of an identified pattern structure beyond the population it was derived in.

## Supplementary material

For supplementary material accompanying this paper visit http://dx.doi.org/10.1017/S0007114518001800. click here to view supplementary material
