# Supplementary material for: Exploratory dietary patterns: a systematic review of methods applied in pan-European studies and of validation studies
Source: Br J Nutr. 2018 Aug 1;120(6):601–11. doi: 10.1017/S0007114518001800 (PMC6137382; doi:10.1017/S0007114518001800)
Supplement: Supplementary file 1 [file S0007114518001800sup001.docx]

Online Supplementary Material

Supplementary Table S1 Details of the search strategy

| #1 (search terms for diet) | (((((diet[All Fields] OR dietary[All Fields]) AND (habit)) OR ((diet[All Fields] OR dietary[All Fields]) AND (pattern[All Fields] OR patterns[All Fields])) OR ((diet[All Fields] OR dietary[All Fields]) AND (quality))) OR (((food) AND (habit)) OR ((food) AND (pattern[All Fields] OR patterns[All Fields])) OR ((food) AND (quality))) OR (((eating) AND (habit)) OR ((eating) AND (pattern[All Fields] OR patterns[All Fields])) OR ((eating) AND (quality))) OR (((nutrition[All Fields] OR nutritional[All Fields]) AND (habit)) OR ((nutrition[All Fields] OR nutritional[All Fields]) AND (pattern[All Fields] OR patterns[All Fields])) OR ((nutrition[All Fields] OR nutritional[All Fields]) AND (quality)))) OR (("western pattern") OR ("prudent pattern") OR ("traditional pattern") OR ("conservative pattern")))  **194941 results** |
| --- | --- |
| #2 (search terms for methods to derive dietary patterns) | (("principal component analysis") OR ("factor analysis") OR ("cluster analysis") OR ("reduced rank regression") OR ("partial least square") OR ("treelet transform"))  **144087 results** |
| #3 (search terms for indices and scores) | ("Healthy eating index") OR ("Mediterranean score") OR ("Dietary guidelines index") OR ("Healthy diet indicator") OR ("diet index") OR ("diet score") OR ("diet quality") OR ("food index") OR ("food score") OR ("food quality") OR ("diet diversity score") OR (“dietary diversity score”) OR ("diet variety score") OR ("dietary variety score") OR ("food variety score") OR (“nutrient index”) OR (“nutrient score”) OR (“Mediterranean adequacy”)  **9217 results** |
| #4 = #1 AND #2 | **5776 results** |
| #5 = #4 OR #3 | **14599 results** |
| #6 (pan-European countries) | Europe* OR Albania[All Fields] OR Andorra[All Fields] OR Armenia[All Fields] OR Austria[All Fields] OR Azerbaijan[All Fields] OR Belgium[All Fields] OR Bosnia[All Fields] OR Herzegovina[All Fields] OR Bulgaria[All Fields] OR Croatia[All Fields] OR Cyprus[All Fields] OR Czech[All Fields] OR Denmark[All Fields] OR Estonia[All Fields] OR Finland[All Fields] OR France[All Fields] OR Georgia[All Fields] OR Germany[All Fields] OR Greece[All Fields] OR Hungary[All Fields] OR Iceland[All Fields] OR Ireland[All Fields] OR Italy[All Fields] OR Latvia[All Fields] OR Liechtenstein[All Fields] OR Lithuania[All Fields] OR Luxembourg[All Fields] OR Malta[All Fields] OR Moldova[All Fields] OR Moldavia[All Fields] OR Monaco[All Fields] OR Montenegro[All Fields] OR Netherlands[All Fields] OR Norway[All Fields] OR Poland[All Fields] OR Portugal[All Fields] OR Romania[All Fields] OR "Russian Federation"[All Fields] OR "San Marino"[All Fields] OR Serbia[All Fields] OR Slovak[All Fields] OR Slovakia[All Fields] OR Slovenia[All Fields] OR Spain[All Fields] OR Sweden[All Fields] OR Switzerland[All Fields] OR Macedonia[All Fields] OR Turkey[All Fields] OR Ukraine[All Fields] OR "United Kingdom"[All Fields] OR UK[All Fields] OR International[All Fields] OR "Europe"  **6806324 results** |
| #7 (multi-country studies) | "Multi-country"[All Fields] OR "Countries"[All Fields] OR "International"[All Fields]  **713970 results** |
| #8 = #5 AND #6 AND #7 | **937 results** |
| #9 (animal studies) | animal studies  **1022269 results** |
| #10 = #8 NOT #9  (exclusion of animal studies) | **878 results** |
| #11 (with filters) | - timespan 1^st^ jan 1990 to 15^th^ jan 2018  - language: English  **853 results** |

Supplementary Table S2 Quality assessment of the included studies

| Author/ Study (Year) | Is the design evident to answer our study question? | Are the subject characteristics sufficiently described? | Is the method of diet assessment described? | Is the diet pattern method well defined and are the details of assessment reported? | Is some estimate of variance reported for the dietary patterns?  (Validity of dietary assessment instrument) | Summary score |
| --- | --- | --- | --- | --- | --- | --- |
| Balder, H.F./  DIETSCAN project including: ATBC NLCS SMC ORDET  (2003) | yes | yes | yes | yes | yes | 10/10 |
| Bamia, C./  EPIC-Elderly study  (2007) | yes | partial | yes | yes | yes | 9/10 |
| Havemann-Nies, A./  US Framingham Cohort SENECA  (2001) | yes | partial | yes | yes | no | 7/10 |
| Iqbal, R. /  INTERHEART study  (2008) | yes | yes | yes | yes | yes | 10/10 |
| Menotti, A. /  Seven Countries Study  (1999) | yes | yes | yes | partial | no | 7/10 |
| Pala, V./  IDEFICS  (2013) | yes | yes | yes | yes | yes | 10/10 |

Supplementary Table S3 Search terms for the additional search of studies, which validated dietary patterns

| #1 (search terms for diet) | (((((diet[All Fields] OR dietary[All Fields]) AND (habit)) OR ((diet[All Fields] OR dietary[All Fields]) AND (pattern[All Fields] OR patterns[All Fields])) OR ((diet[All Fields] OR dietary[All Fields]) AND (quality))) OR (((food) AND (habit)) OR ((food) AND (pattern[All Fields] OR patterns[All Fields])) OR ((food) AND (quality))) OR (((eating) AND (habit)) OR ((eating) AND (pattern[All Fields] OR patterns[All Fields])) OR ((eating) AND (quality))) OR (((nutrition[All Fields] OR nutritional[All Fields]) AND (habit)) OR ((nutrition[All Fields] OR nutritional[All Fields]) AND (pattern[All Fields] OR patterns[All Fields])) OR ((nutrition[All Fields] OR nutritional[All Fields]) AND (quality)))) OR (("western pattern") OR ("prudent pattern") OR ("traditional pattern") OR ("conservative pattern")))  **171368 results** |
| --- | --- |
| #2 (search terms for methods to derive dietary patterns) | (("principal component analysis") OR ("factor analysis") OR ("cluster analysis") OR ("reduced rank regression") OR ("partial least square") OR ("treelet transform"))  **105212 results** |
| #3 (search terms for indices and scores) | ("Healthy eating index") OR ("Mediterranean score") OR ("Dietary guidelines index") OR ("Healthy diet indicator") OR ("diet index") OR ("diet score") OR ("diet quality") OR ("food index") OR ("food score") OR ("food quality") OR ("diet diversity score") OR (“dietary diversity score”) OR ("diet variety score") OR ("dietary variety score") OR ("food variety score") OR (“nutrient index”) OR (“nutrient score”) OR (“Mediterranean adequacy”)  **4535 results** |
| #4 (search terms for  reliability/validity) | (“Validity”) OR (“Valid”) OR (“Validation”) OR (“Reliability”) OR (“Reliable”)  431656 |
